# Affective Neuroscience: Implications for the Jungian Self and Archetypes

**DOI:** 10.1111/1468-5922.70086

**Published:** 2026-09-03

**Authors:** Oliver Dale, Andrew John Howe

**Affiliations:** ^1^ Littlehampton UK; ^2^ London UK

**Keywords:** affective core, affective neuroscience, analytical psychology, archetypes, cognitive neuroscience, Jung, noyau affectif, archétypes, neurosciences affectives, neurosciences cognitives, Jung, psychologie analytique, Affektiver Kern, Archetypen, affektive Neurowissenschaft, kognitive Neurowissenschaft, Jung, Analytische Psychologie, nucleo affettivo, archetipi, neuroscienze affettive, neuroscienze cognitive, Jung, psicologia analitica, аффективное ядро, архетипы, аффективная нейробиология, когнитивная нейробиология, Юнг, аналитическая психология, : núcleo afectivo, arquetipos, neurociencia afectiva, neurociencia cognitiva, Jung, psicología analítica, 情感核心, 原型, 情感神经科学, 认知神经科学, 荣格, 分析心理学

## Abstract

Archetypes are central to Jung’s model of the mind and his concept of the Self. He observed that patients with psychosis demonstrated cultural content in their hallucinations and delusions that were not learned, leading him to define archetypes as impersonal unconscious patterns. Although Jung’s description of archetypes evolved over time, the core idea of inherited, universal patterns in psychological and cultural phenomena has remained consistent. The paper aims to provide an overview of affective neuroscience and Panksepp’s brain hierarchy model, to summarize contemporary archetypal thinking and to explore what Panksepp’s affective neuroscience can add to our understanding of archetypes. We end with examples of how this novel combination of theories can play out in the therapy room and in our day‐to‐day lives.

## Abstract (fr)

Les archétypes occupent une place centrale dans le modèle psychique de Jung et dans sa conception du Soi. Il a observé que les patients atteints de psychose présentaient, dans leurs hallucinations et leurs délires, des éléments culturels qui n’étaient pas acquis par l’apprentissage, ce qui l’a conduit à définir les archétypes comme des schémas inconscients impersonnels. Bien que la conception des archétypes chez Jung ait évolué au fil du temps, l'idée fondamentale de motifs hérités et universels dans les phénomènes psychologiques et culturels est restée inchangée. Cet article a pour objectif de donner un aperçu des neurosciences affectives et du modèle de hiérarchie cérébrale de Panksepp, de résumer la réflexion contemporaine sur les archétypes et d'examiner ce que les neurosciences affectives de Panksepp peuvent apporter à notre compréhension des archétypes. Nous terminerons par quelques exemples illustrant comment cette combinaison novatrice de théories peut se traduire en consultation et dans notre vie quotidienne.

## Abstract (de)

Archetypen sind zentral für Jungs Modell des Geistes und seines Konzeptes des Selbst. Er beobachtete, daß Patienten mit Psychosen in ihren Halluzinationen und Wahnvorstellungen kulturelle Inhalte aufwiesen, die nicht erlernt waren, was ihn dazu veranlaßte, Archetypen als unpersönliche, unbewußte Muster zu definieren. Obwohl sich Jungs Beschreibung von Archetypen im Laufe der Zeit weiterentwickelt hat, ist der Kerngedanke ererbter, universeller Muster in psychologischen und kulturellen Phänomenen unverändert geblieben. Dieser Artikel bietet einen Überblick über die affektive Neurowissenschaft und Panksepps Modell der Gehirnhierarchie, faßt das zeitgenössische archetypische Denken zusammen und untersucht, welchen Beitrag Panksepps affektive Neurowissenschaft zu unserem Verständnis von Archetypen leisten kann.

Zum Schluß zeigen wir Beispiele dafür, wie sich diese neuartige Kombination von Theorien im Therapieraum und in unserem Alltag auswirken kann.

## Abstract (it)

Gli archetipi sono centrali nel modello junghiano della mente e per il suo concetto di Sé. Jung aveva osservato che i pazienti psicotici mostravano contenuti culturali, che non erano stati appresi, nelle loro allucinazioni e nei deliri, e questo lo ha portato a definire gli archetipi come pattern inconsci impersonali. Sebbene la descrizione che lui ha fatto degli archetipi si sia evoluta negli anni, l'idea centrale di pattern universali ereditari per i fenomeni psicologici e culturali è rimasta costante. Questo articolo si prefigge di offrire una panoramica sulle neuroscienze affettive e sul modello gerarchico del cervello, secondo Panksepp, di sintetizzare il pensiero contemporaneo sugli archetipi e di esplorare in che modo le neuroscienze affettive di Panksepp possano aggiungere qualcosa alla nostra comprensione degli archetipi. Concludiamo con esempi di come questa combinazione tra teorie po avere un ruolo nella stanza d'analisi e pure nella vita quotidiana.

## Abstract (ru)

Архетипы занимают центральное место в юнговской модели психики и его концепции Самости. Юнг заметил, что в галлюцинациях и бреде пациентов в психотическом состоянии появляется неизвестное им культурное содержание, что навело его на мысль об архетипах как безличных бессознательных паттернах. Хотя определение архетипов Юнгом со временем эволюционировало, его центральная идея о наследуемых универсальных паттернах в психологических и культурных явлениях оставалась неизменной. Цель статьи – дать представление об аффективной нейробиологии и модели иерархии мозга Панксеппа, обобщить современное состояние теории архетипов и проанализировать, что может добавить к нашему пониманию архетипов концепция аффективной нейробиологии Панксеппа. В заключение мы приводим примеры того, как эти два подхода могут сочетаться в терапевтическом кабинете и в нашей повседневной жизни.

## Abstract (es)

Los arquetipos y su concepto del Self son centrales en el modelo de la mente de Jung. Jung observó que los pacientes con psicosis mostraban en sus alucinaciones y delirios contenidos culturales que no habían sido aprendidos, lo que le llevó a definir los arquetipos como patrones inconscientes e impersonales. Aunque la descripción de Jung de los arquetipos evolucionó con el tiempo, la idea central de patrones universales y heredados en los fenómenos psicológicos y culturales se ha mantenido constante. El artículo tiene como objetivo ofrecer una visión general de la neurociencia afectiva y del modelo de jerarquía cerebral de Panksepp, resumir el pensamiento arquetipal contemporáneo y explorar qué puede aportar la neurociencia afectiva de Panksepp a nuestra comprensión de los arquetipos. Concluimos con ejemplos de cómo esta novedosa combinación de teorías puede aplicarse en la consulta terapéutica y en nuestra vida cotidiana.

## Abstract (zh)

原型是荣格心灵结构和“自性”概念的核心。他观察到精神病患者在幻觉与妄想中呈现出他们从未学习过的文化内容, 由此提出原型是‌非个人的无意识模式‌。尽管荣格对原型的描述随时间不断演变, 但其观点的核心部分从未改变, 即在心理与文化现象中存在遗传的、普遍的模式。本文旨在综述‌情感神经科学‌与‌潘克塞普的脑层级模型‌, 梳理当代关于原型的理论思考, 并探讨潘克塞普的情感神经科学如何深化我们对原型的理解。最后, 文章将举例说明这一创新性的理论融合如何应用于心理治疗实践及日常生活。

## Introduction

The word “archetype” is accepted as common parlance; however, such common usage obscures the considerable shift in human psychological theory it represents. At the time of Jung’s first description of the concept, it was believed that humans were born as a “blank slate”, the *tabula rasa* of John Locke’s enlightenment philosophy, which assumed that at birth our minds are empty vessels waiting to be filled (Porter, 2003). Jung dismissed the tabula rasa theory, arguing that a newborn interacts with sensory stimuli in specific ways that indicate inherited instincts and preformed patterns (Jung, 1954a, para. 136).

The concept of archetypes is central to the Jungian model of the mind and at the heart of the Jungian Self. Jung first considered the concept of archetypes while working as a newly qualified doctor at the Burghölzli psychiatric hospital under Eugen Bleuler. Here he observed that patients with psychosis presented cultural knowledge in their hallucinations and delusions that was not learned (Jung, 1952, para. 151). His first definition, made in 1919, separated out the impersonal unconscious nature of archetypes (archetypal images) from conscious representations, as in works of art created by humanity (Jung, 1919). Over the course of his writings, Jung incorporated philosophy, religion, mythology and science into his theories.

Some consider Jung’s description of archetypes as inconsistent (Knox, 2003, Chapter 2). However, it is understandable that over time he would develop, expand and change his thinking on the subject. Post‐Jungian development of archetype theory has similarly been unable to arrive at a unified definition (Roesler, 2019). What appears to have remained consistent, however, is this central idea of inherited, universal patterns observable in psychological and cultural phenomena.

In this paper, we aim to explore what the field of Affective Neuroscience might add to our understanding. We first summarize some key contemporary archetypal thinking before giving an overview of Affective Neuroscience and Panksepp’s three‐layer nested brain hierarchy (Panksepp, 2014).

## Archetype

Archetypes are a priori structures, formless in themselves and present in the unconscious of every psyche. They are active and continually influence our thoughts, feelings and actions (Jung, 1954b, paras. 154–155). They can be understood via archetypal images, which are different expressions of archetypes related to personal and cultural factors. Jung first considered his concept of archetypes while working as a newly qualified doctor at the Berghölzli psychiatric hospital under Bleuler. Here he observed that patients with psychosis revealed cultural knowledge in their hallucinations and delusions they would not have otherwise known (Jung, 1952, para. 151). A dictionary of analytic psychology describes archetypes as: “The inherited part of the psyche; structuring patterns of psychological performance linked to instinct; a hypothetical entity irrepresentable and evident only through its manifestations” (Samuels, Shorter & Plaut, 1997, p. 26). Samuels later comments that archetypes have a numinous quality and are linked with affect.

Samuels’ definition is notable for its link to instinct, a biological term, while considering archetypes as hypothetical entities that do not exist in the physical world. Crucially, the word “numinous” introduces a spiritual dimension. We therefore have a psychological process that appears at first glance to contain both biological and non‐biological aspects; it is both corporeal and non‐corporeal.

Knox offers a classification of archetypes which provides us with a means of differentiating four aspects of archetypes by giving four categories:

- biological entities in the form of information which is hardwired in the genes, providing a set of instructions to the mind as well as to the body
- organizing mental frameworks of an abstract nature, a set of rules or instructions but with no symbolic or representational content, so that they are never directly experienced
- core meanings which do contain representational content and which therefore provide a central symbolic significance to our experience
- *metaphysical entities which are eternal and are therefore independent of the body*. (Knox, 2003, p. 23).

Whilst Knox’s classification provides a framework, it leaves open the question of whether observations of archetypes are a manifestation of biological, psychological, or metaphysical processes, or if they are a mixture of all the above.

## Archetype as Image Schema

Whilst offering the above classification, Jean Knox’s (2003) explanation of archetypal communication heavily leans on the concept of image schemas. Part of her argument rests on the simple proposition that there is no way the human genome can encode the amount of information traditional archetypal theory supposes. Her explanation of archetypal phenomena is that they emerge out of the implicit learning that accompanies developmental processes as opposed to some kind of genetically encoded internal and instinctual model. She considers much of this learning as universal and imbued with what we collectively share as organisms. In our opinion, such an understanding could potentially explain archetypes not as phenomena in themselves but as epiphenomena arising out of universal developmental processes.

Knox’s argument appears to locate the biological component of archetypes primarily in the bodily and relational reality of existence as opposed to the body itself. Whilst she leaves room for the other categories of archetypes, her theorizing focuses on this one category. The issue with this is that the narrowing of the role of a bodily, living archetype leaves the body lifeless as it loses its role as a mediator between the ancient and the present, which archetypal phenomena illustrate. Furthermore, the lifelong engagement with the nurturing and driving aspects of the Self and its archetypal reservoir is lacking in such an understanding. We think there is something uninspiring lifeless in such a limited interpretation. We believe that the contribution of Affective Neuroscience, outlined in this paper, makes an important contribution to understanding this lifelong expression of archetypes.

## Bodily Archetype and the Humble Reflex

To illustrate the role of the body, even at the most basic level of an archetype, the reflex is an inherited behaviour precipitated by environmental cues. Past evolutionary inheritance is present in humanity today, “encoded” in the body and brought to life in the present moment. The most obvious example is the rooting reflex, present at 28 weeks of gestation. This mediates the infant’s move towards the nipple, stimulated by either the smell of milk or being touched on the cheek near the mouth. Taken with other primitive reflexes, present at birth and without the opportunity for psychological or cultural mediation, reflexes endow the infant with a set of instructions that mediate survival.

Of course, we need to go further to explain the richness of archetypal expression. However, these reflexes point to a universal and ancient method of instruction, indicating that a genetically determined precedent is possible. Furthermore, they point to an interaction between the infant and the environment, and they open up the possibility of image schemas arising out of the interaction as per Knox’s proposal. To quote Knox, the image schema is the “first stage of a process whereby the brain constantly sorts and classifies sensory information into meaningful conceptual categories” (Knox, 2003, p. 55).

## The Role of Epigenetics

Taking this a step further, Goodwyn (2020) points out that the genome is now thought to contain 30,000 more genes than it was believed we had at the time of Knox’s writing. It is more difficult to define a gene than one might think, but there are probably more than 100,000, maybe as many as 300,000 productive segments of DNA (Salzberg, 2018). This tenfold increase expands the possibilities of what is “hard wired” into genetics.

Perhaps more importantly, however, Goodwyn takes us beyond the simple DNA sequence by tackling Knox’s supposition that the “archetypal” influence of genes ends at birth by pointing out that the genetics of an individual and the life of the individual are in a complex interaction throughout the life cycle through epigenetic processes.

Epigenetics studies heritable traits, or changes in cell function, that happen without changes to the DNA sequence. These processes refer broadly to regulating the expression of DNA sequences, often mediated by adding methyl groups to “promoter” areas of DNA. A methyl group is a carbon atom with three hydrogen atoms attached to it, and this group can attach itself to specific sections of DNA to turn on or off the expression of the subsequent protein which that section codes.

Goodwyn gives the example of the lactose operon in E. coli to illustrate how this relates to an archetype. An operon is a functioning unit of DNA containing a cluster of genes under the control of a single promoter, in this case, metabolizing lactose. The E. coli bacteria usually metabolizes glucose. However, if the outside environment contains only lactose, the operon will activate, and it will metabolize lactose instead. E. coli never uses this operon if it remains in a glucose‐rich environment and may go its entire life without using it. However, the moment the environmental pressure presents itself, this potential is activated is activated.

Considering the operon as a basic biological concept, Goodwyn approaches the archetype in a similar vein: an inherited mechanism leading to a predetermined action but only in the context of a specific environmental condition. Goodwyn’s overall argument is that we can view archetypes as genetically inherited, if we take account of epigenetics. In this way, an archetypal response may be inherited, lie dormant and only be activated once certain conditions are met.

## Archetype as “Neural Code”

Using recent developments in neuronal code biology, Major (2021) considers archetypes as biological algorithms drawn from the evolutionary past. Neural codes are forms of computation, a set of rules, which facilitate communication. In this case, communication is between our senses on one side and emotional responses on the other. Universal processes, i.e. archetypes, govern its facilitation. Major describes how when we surprise ourselves by acting in ways that do not coincide with our conscious attitude, we are, instead, acting based on an unconscious a priori emotional and behavioural algorithm. This algorithm reflects an inherited “biological wisdom” (2021, p. 5), which may, for example, be focused on survival and personal growth.

## Three Components of Bodily Archetypal Information

Drawing on code biology, Vedor (2023) frames archetypes as innate structures that prepare us for situations we may encounter in the real world. He uses Jung’s analogy to archetypes being an old riverbed, where water may run again. He presents a triadic model of archetypes and describes the structural, regulatory, and representational aspects. Structural are the deepest and most embedded archetypes, unconsciously guiding experiences and behaviours. At their most basic level, structural archetypes are found within our genetic code. Using the riverbed analogy, they are river courses themselves without water. Regulatory archetypes are described as sluice gates along the river, regulating the flow, “guiding” timely archetypal expression, preventing us from becoming overwhelmed. They determine when and how the structural archetypes are expressed. Finally, representational archetypes are manifest content shaped by the preceding two archetypes. They are the actual river flow, the present event. Overall, Vedor aims to show that while the genome provides a blueprint for development, it is not the sole determinant, and archetypes are more than just genetic code as they arise out of an interaction between genetics and environment.

From a bodily point of view, we therefore have three distinct components of archetypal communication to consider:

- **Reflex:** basic, determined by the gene sequence giving rise to highly automatic bodily responses
- **Epigenetic**: more complex, determined by the variable expression of gene sequences influencing more global responses of the organism in response to environmental cues
- **Neural code:** highly complex, determined by the expression of our genetic material (genetic and epigenetic) to give a set of algorithms which guide life

To explore these three components, we turn to Affective Neuroscience.

## Affective Neuroscience

Using a range of approaches, Panksepp coined the term Affective Neuroscience (AN) to delineate an area of research focused on cross‐species brain science (Panksepp, 2004). Through the application of Deep Brain Stimulation, preferential breeding in rats, pharmacological challenges, and studying the effects of brain lesions on mammals and humans, a model of affective systems has been described, which is present, in various degrees, across the chordate animal kingdom.

Humans share a common cerebral ancestry with all chordate animals. The most unique part of the human brain is our enlarged dorsolateral prefrontal cortex, but apart from that, the basic neural architecture is found in all other vertebrates’ brains.

One way of broadly classifying brain structures is to draw a distinction between cortical and subcortical regions with the more recent cortex containing the frontal, parietal, temporal and occipital lobes and the older subcortex being much more diverse, containing the brain stem, cerebellum and limbic structures. For our purposes, we are glossing over considerable complexity (some consider the limbic system as the paleomammalian cortex for instance). Broadly speaking, however, we will refer to cortical and subcortical regions hereon.

To understand affective systems and how they operate within our broader psyche, our focus must shift from the cortex to subcortical structures. Along with Panksepp, Alcaro and Carta (2017) describe the subcortex as the “first neuro‐evolutional layer of the mind … the affective core of the Self”. An understanding of this layer is crucial for Jungian theory as it is this older area, as distinct from the more modern cognitive one, which deals with affect.

In human cases of hydranencephaly (where the cerebral cortex and higher limbic areas are destroyed before birth due to cerebral infarction during gestation), leaving subcortical function only, there remain observable forms of consciousness based on responses to positive and negative affective states including expressing pleasure or “fussing” as well as showing indications of learning and purposeful behaviour. This is a crucial point. You do not need a cortex for conscious life; you can be conscious with just a functioning subcortex.

Furthermore, experimental observations seem to indicate that subcortical and brain stem structures mediate three categories of affective states:

- **Homeostatic/Visceral affects**: an awareness of internal bodily states perceived as moods or feelings. These visceral affects effect homeostatic mechanisms of the body such as the endocrine and autonomic system, also called the “internal milieu”.
- **Instinctual/emotional affects**: refer to a disposition to act through avoiding or approaching certain situations. Typically, these are negative or positive affects, which motivate the organism to move towards or away from the stimulus.
- **Sensorial affects**: give perceptual experiences an affective value, such as the pleasure of warmth or the unpleasantness of a startling sound. These subcortical structures include specific nuclei within the brainstem which direct motor activity, such as a startle response.

Operating across these different affective categories, brain stimulation studies indicate that the activation of specific brain stem areas modifies our subjective states, generating distinct affects. These affects align with various associated behaviours. In creatures with a brainstem seven fundamental emotional dispositions can be found (Panksepp, 2014):

- **SEEKING system:** moves the animal to look for resources.
- **RAGE system:** moves the animal to compete for or defend resources.
- **FEAR system:** moves the animal to avoid danger.
- **LUST system:** identifies potential mates and reproduces.

Panksepp (2014) distinguishes the mammalian brain by its inclusion of the primary affective states of PANIC, PLAY, CARE, suggesting these systems underpin social bonding.

- **CARE system:** moves mammals to care for offspring.
- **PANIC/GRIEF (separation distress) system:** promotes social bonding.
- **PLAY system:** moves mammals to physically play to learn and build social bonds.

Some of these systems are described as being aversive in that they motivate the organism away from the stimulus through the flight or fight system, whilst the others promote an approach towards the stimulus.

What Panksepp and colleagues have described is a set of seven discrete motivational systems which form the bedrock of our affective life. As affective states, they are a primary unthinking form of experience that, in their raw, unmodified form, are innate motivational systems that play a key role in survival and development from birth onwards. Accordingly, Alcaro, Carta and Panksepp (2017) consider these systems as “archetypes as such” in that, whilst shared collectively, they are primary organizations that originate within the individual and reveal themselves through action and affect. Governing actions in response to changes in our internal and external environments, they also fit the description of a neural code.

## “Layered Consciousness”

An implication of the affective core is that the term unconscious becomes problematic. In common parlance, unconscious means to be unaware. However, it may be helpful to consider human awareness as having two distinctive aspects derived out of affective and cognitive functions. This resonates with Antonio Damasio’s (2000) description of “core” and “extended” consciousness. “Core” describes the individual’s feeling experience at one moment in time, and “extended” consciousness is a symbolic representation derived out of cognition.

Representing a point in time, extended consciousness relies upon the capacity to store, to manipulate and communicate information based on symbolic representation, as opposed to the core, which is instinctual, automatic and in the moment. Therefore, awareness derived from “feeling” arises from a primordial non‐cognitive aspect of the psyche that is immediate and influenced by both internal and external worlds, while “thinking” is a cognitive aspect that emerges from affect. Such an understanding is in keeping with the Jungian idea of there being two locations of consciousness—Self and Ego. Specifically Jung notes that there must be a *“an unconscious ego … a second consciousness”* (Jung, 1969, para. 385). The idea of two or more centres of conscious awareness was furthered in post‐Jungian work, for example, by West who commented on the narrowness of Jung’s idea of ego, noting that it does not correspond totally with conscious awareness. Indeed, there may even be many “I’s” that become consciously aware when we consider the psyche as a whole (West, 2008). In the Black Books (Jung, 2020), e.g., “The Seven Sermons to the Dead”, Jung notes that it was not he who spoke to the dead but that it was Philemon talking through him, or Jung’s interactions with at least eight distinct identities from his unconscious. Taking this a step further, Alcaro and colleagues draw on Tulving’s three‐layered classification of consciousness (Tulving, 1985). Although it is beyond the scope of this paper to be drawn too far into what we mean by cognition, we will need a rough outline for our purposes here. In short, Tulving describes the following layers of consciousness:

- **Anoetic:** an unthinking form of consciousness which is affectively intense without being known. It is present across the animal kingdom and located in the subcortical areas of the brain. Memory at this level is procedural (heavily related to the cerebellum) and is bound to the current situation. We posit this to be the archetypal layer.
- **Noetic:** a thinking form of consciousness linked to exteroceptive perception and cognition. It allows for a cognitively driven awareness and manipulation of the being’s immediate environment. It is a rudimentary form of cognitive consciousness and incorporates learning and planning. It is common in animals and observable in young infants, sometimes referred to as “primary consciousness”.
*“Noetic consciousness allows an organism to be aware of, and to cognitively operate on, objects and events, and relations among objects and events”* (Tulving, 1985, p. 12)
  i.e., this requires a semantic and, therefore, symbolic form of memory.
- **Autonoetic:** A form of conscious awareness that allows self‐reflection. This is a much more sophisticated form of awareness that involves language, narrative, and the application of symbolism, sometimes referred to as secondary consciousness. Tulving describes how this form correlates with episodic memory:
  “*When a person remembers such an event, he is aware of the event as a veridical part of his own past existence. It is autonoetic consciousness that confers the special phenomenal flavour to the remembering of past events, the flavour that distinguishes remembering from other kinds of awareness, such as those characterizing perceiving, thinking, imagining, or dreaming.”* (Tulving 1985, p. 12)

## Body as “Subject”, Body as “Object”

To understand the layered consciousness further, we need to draw a crucial distinction between the way the cortex and the subcortex “experience” the body. Solms and Panksepp (2012) describe a fundamental “dual aspect” of the mind’s representation of the body which they refer to as the interoceptive Id and the exteroceptive Ego. We hope to show how such a description fits far better with the Jungian two‐centre model of Self and Ego.

The interoceptive and subcortical Id, or Affective Core, is preoccupied with the internal milieu of the organism. This autonomic body (autonomic refers to “self‐government”) includes the various structures and processes designed to monitor and regulate the body automatically to maintain homeostasis. Panksepp and Solms refer to this as the “internal body” and whilst it might function independently, it can, and does, arouse the external body through affect when a threshold is crossed. Alcaro et al. describe an anoetic proto‐conscious feeling emerging as a neurodynamic “wave’s crest” from a continuous flux of diffuse arousal without focus or intent (Alcaro, Carta & Panksepp 2017, p. 8).

The exteroceptive Ego, however, represents the body through the somatotopic maps of the cortex. This includes various senses that give a cognitive experience of the body and the world around it, such as external temperature, sound, movement, pain, etc. This, they refer to as the “external body”. In contrast to the internal body, this external body, and our connection to it, is learnt through experience. Primed for learning via a pre‐prepared somatotropic and sensory map of the body and world around it, the senses are organized into an ego, built out of the “wave‐crests” above. As such, the ego is extended out of the affective core, integrated with it, and created following birth. This means that with pinpoint accuracy, the thumb is not only anatomically represented, but the infant discovers it to be “their” thumb!

Solms and Panksepp (2012) stress that the internal body is not represented as an image or an object of perception but as a background sense of being. It is an awareness through feeling, and its phenomena are experienced as affects with positive and negative valences, which are focused on survival and reproductive success. The foci are organized around the primary affect systems described above. This gives the organism an undertone of affect, very much in the background, colouring everything, but occasionally coming to the foreground.

Interoceptive consciousness is phenomenal: it “feels like” something, and these phenomenal states of the body‐as‐subject are experienced affectively as positively and negatively valenced states. Their valence is determined by how changing internal conditions relate to the probability of survival and reproductive success (Solms & Panksepp, 2012, p. 156). Although functional at birth, the interoceptive, anoetic, affective core can be modified by learning. However, this remains at the procedural level and so a meta‐sense is built up much like a distinction between weather, the present condition and climate, the average over time.

The exteroceptive Ego is different: it is precise and representative. Returning to our now discovered thumb, it is symbolically and cognitively represented (and we mean this literally) within the motor areas of the cortex. Along with all the other cognitive representations of the body and the world it inhabits, it is symbolically “presented” in space and time across the cortex. To quote Freud, the ego is first and foremost a bodily ego (1923).

## The Nested Brain Hierarchy

Solms and Panksepp (2012) describe this interconnected representation through the Nested Brain Hierarchy (NBH) model. We have modified this graphic model to represent the archetypal collective material as larger and vaster than the ego (Figure 1). The premise is that the most sophisticated form of conscious experience is founded upon a basic affect modified through different layers of the brain, with the limbic system being the second layer and the neocortex the third. A conscious idea contains an emergent affect transformed through a supervenient process.

**Figure 1:**
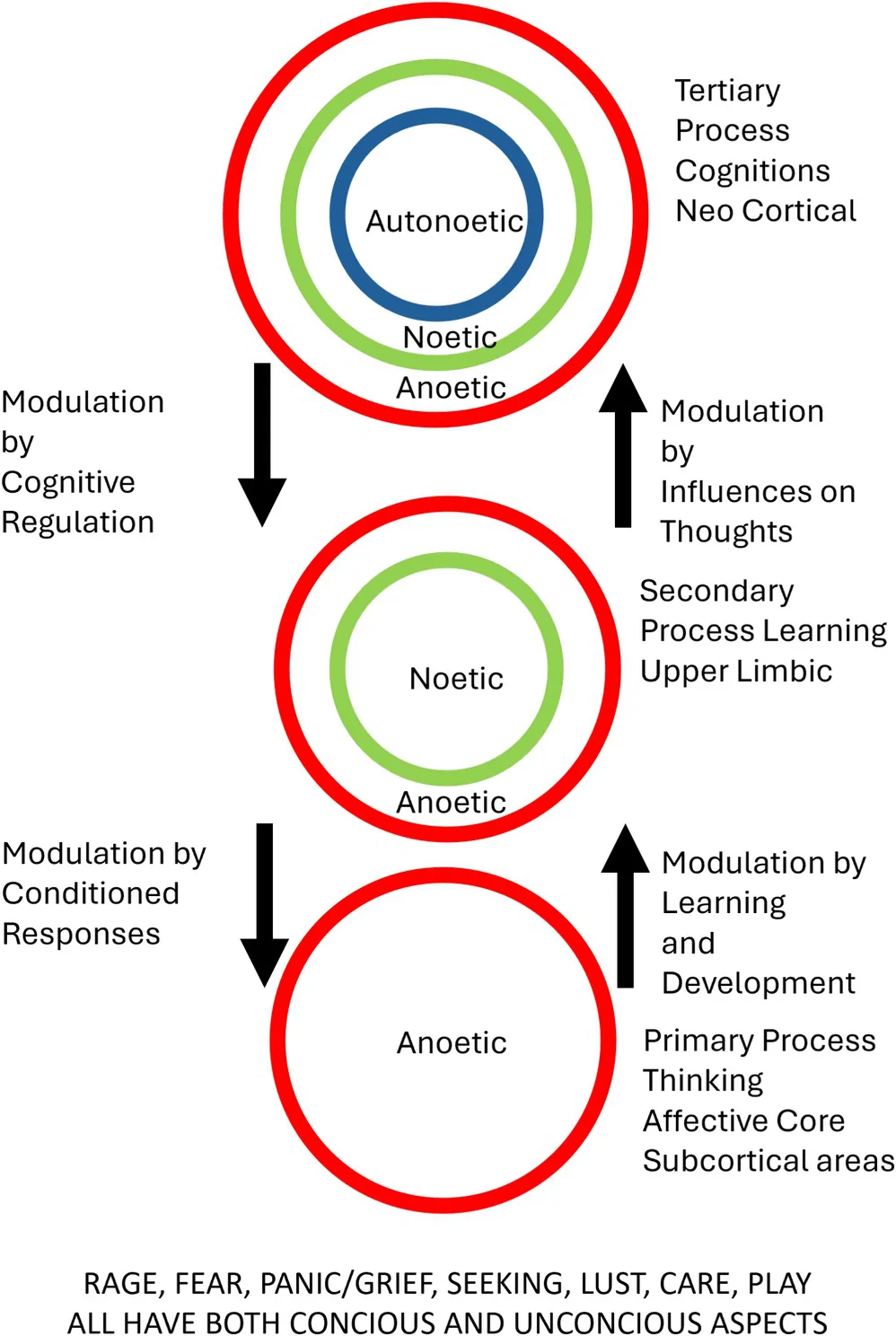
The Nested Brian Hierarchy adapted by the authors from Solms and Panksepp (2012)

## Summary and Proposition

Jung’s archetypal theory sought to integrate a biological and evolutionary understanding of psychological phenomena that he had observed at the Burghölzli Hospital. His observations led him to consider archetypes as real, organic entities whose expression was influenced by the external environment. A gap, however, in this theory was that the creation of archetypal imagery lacked a credible biological explanation. Knox’s attempt to explain such phenomena through image schema, whilst sound, is considered insufficient as an explanation.

Building upon the work of Alcaro, Carta and Panksepp, we argue for incorporating Affective Neuroscience into archetypal theory. Affective Neuroscience’s description of an interconnected model of subcortical affective awareness as a foundation to an upper limbic and neocortical form of cognition fits very well with a model of the mind containing two centres of awareness with an archetypal Self and self‐reflective ego.

## Thunder—A Demonstration of Application

If we consider the multisensory experience of a thunderstorm, it encompasses loud bangs, flashes of light, spears of lightning striking the ground, rain, and wind. We can see how this would represent a threat to life and necessitate a response from the animal to improve its chances of survival. This may trigger responses from the FEAR and PANIC/GRIEF or even RAGE systems. Drawing on Major’s theory (2021), these responses would be archetypal and represent biological wisdom to survive.

Using the example of a dog with a phobia of thunder (Gruen & Sherman, 2012) we can consider this model further. We have chosen to use an animal example to emphasize the cross‐species and evolutionary underpinnings of archetypes and to eliminate any advanced tertiary‐level thinking which could muddy the waters of the development of our argument. For the first two years of life, the dog in the case references responded to thunderstorms with whining, pacing and other fearful responses alleviated by attention and care from its owners. From an NBH viewpoint, this carer’s response could be seen as secondary level noetic learning and modulation of a primary affective response and activation of the CARE system. The dog’s primary level response to flee in fear was subdued.

What if the owners were not always present when thunderstorms occurred? One can imagine the dog responding by scratching the doors in an attempt to escape. Indeed, this is exactly what happened and what led to the dog's referral to a veterinarian, the response arising out of the wish to flee in fear because this environmental factor occurred in the absence of the modulating presence of the owners. In the absence of the CARE system the dog is left with the FEAR and PANIC/GRIEF only.

We now come to a human response to a thunderstorm. When we are young, it is understandable that we may respond with archetypal fear to such an event. If our caregivers provide a caring environment and help us understand that we are safe, we quickly learn not to fear what is fearful at an instinctual level. We do not fear because our caregiver makes us feel safe, and this becomes incorporated through learning. This can be understood from a purely secondary level process, as noted in our example with the dog. However, as humans, we can also reflect and add language and metaphor to our experiences at the autonoetic tertiary level. The thunderstorm can then become *like* something else, it can generate feelings and images that we give names to and express. We can abstractly associate other memories that may have no direct link to the storm itself, which can form the basis of mythology. Transcendent across cultures and time, our thunder gods can take human form and, in our reflections, we can see a thunderstorm to symbolize aspects of human behaviour, with all our ranges of emotions.

The creation of stories and mythology recruits higher‐level cortical functions linked to deeper structures and experience. The responses and images all come from the same underlying archetypal layer, but the results will differ based on a multitude of environmental factors. For example, one may draw comparisons between the rage of an abusive father and the experience of thunder. An experience of one would influence the other, and therefore, a behaviour, experience or image resulting from thunder could have a completely different meaning, coloured by the quality of care that was received.

## Clinical Implications

It is worth reflecting on the clinical implications of what we have outlined here. Whilst lacking cognition, the anoetic part of our psyche is powerful, deterministic and affective in nature. People become aware of their “unconscious”, and with luck and enough good care, come to learn how to modulate the intense affective experience it contains. Such modulation integrates affect and cognition to create a thinking function grounded within the body and environment around it.

Our use of the thunderstorm illustrates key observable phenomena marking that journey to thinking. Using the idea of a neural code set by affective systems we are given a way of understanding what it means to be triggered. We all have areas of greater and lesser top‐down modulation. Where it is lesser, the affective system has greater sway and you might see a complex response where archetypal and personal materials are combined, yet still not fully integrated; where it is absent, the affective system has total control, and we witness a more “raw” and unmodified archetypal expression. Such an experience catches the subject unaware or may even feel alien to the subject. To an observer, the subject can seem both powerful and out of control. To a degree, caregivers can act to modulate on the behalf of the cared‐for. With time, such modulation may become incorporated into their psyche, culminating in genuine integration of affect and cognition lowering the propensity to being triggered. This seems to give a modern appreciation to the idea of Bion’s container‐contained. A clinical example could be cancellations of sessions by the therapist. When done in a planned manner, the patient may have more cognitive top‐down control of their responses, feeling a sense of rejection or abandonment but being able to modulate their emotional response. A last‐minute cancellation may lead to a more affective response with the therapist unavailable to help modulate the surge of affective material.

Given the notion of the timelessness of affect and the fact that in the absence of cognitive modulation, affect can be experienced as infinite, such experiences are overwhelming to both patient and therapist. It is this uncontained experience that overwhelms the capacity to think and is inherently confusing. To be clear, individuals do not necessarily “know” when they are overwhelmed by the affective system because the ability for cognitive modulation has become limited.

In time, the storm will pass, and the “wave crest” subsides, meaning the capacity for cognitive modulation recovers. Regretful, the patient may recognize that the proverbial thunderbolt arising toward them from the therapist did not warrant such an overwhelming ATTACK and FEAR response. Such a realization might trigger a PANIC/GRIEF response and a fear of abandonment.

Crucially, we might understand that the person in front of us is experiencing us as a very powerful transferential figure who is not the representation of an actual figure from the past, but more a representation of an absence of a carer able to contain an affective experience in the past. To get such an understanding, we need to deploy our CARE system, exploiting the PANIC/GRIEF system to maintain the relationship. In doing so, we create the opportunity of PLAY, promoting the development of cognitive modulation through interaction and shared meaning‐making.

The task of the therapist is to maintain their capacity to think and therefore digest the experience on behalf of the patient. Such experiences, however, are challenging, and given the nature of the communication, the therapist may become overwhelmed. It therefore takes considerable practice for the therapist to recognize the signs that this is a mutual experience, and they are in the analytic vas together.

To our cognitive selves, the affective core feels “other”. When the thunderstorm has passed, we might even shake our heads and ask, “silly me, did I really need to get so upset?”. In doing so, we are using the CARE system to manage ourselves. In the same way it is common for patients to be gripped by feelings that they don’t understand, the shame of which can be a barrier to owning them. When it is safe and shame is less of a problem, one might hear a patient apologize for being so angry with you. They may even say, I don’t understand why, it just seemed I needed to be. This, though, is often a statement that comes from successful navigation of the storm and a step toward further integration. Typically, this requires an interpersonal experience in order to develop that intrapersonal relationship between the affective core and the ego.

For the Jungian‐minded therapist, we are exploiting our relationship to set the culture for such an intrapersonal relationship to develop. This is not always achievable, and the softer “silly me” type of response is not reached, even with help. This leaves affective core and ego alienated to such a degree that the cognitive self is always buffeted by the powerful affective storms, leaving an ever‐traumatized person feeling hopeless, powerless and disenfranchised from themselves. A case series explored in *Self, ego and suicide* (Dale, 2022) explores this relationship in further detail, but ultimately, this integration is a lifelong individuation process. It is not simply a childhood developmental task. Hence, the affective core and its archetypal material remain very much alive throughout our lives, requiring lifelong learning and modulation, sometimes necessitating therapeutic assistance.

Therapeutic progress is marked by developing the ability to be curious so that the cognitive I can peer down and recognize that there is this part of them, which is powerful, outside of their control, but ineffably a part of them. The process of therapy and the therapeutic relationship are tasked with facilitating that union.

## Summary

The paper explores the relationship between the unconscious affective experiences and cognitive processes and their bearing on the therapeutic process, emphasizing the importance of integrating affect and cognition for effective therapeutic outcomes and individuation. In this paper, whilst we have paid particular attention to the concept of archetypes through the lens of Affective Neuroscience we want to underline that Affective Neuroscience does not offer a comprehensive explanation for it. The field of Affective Neuroscience clearly has a bearing on our understanding of archetypes but can only provide a partial explanation in our view.

In conclusion we would summarize the following:

- **Understand the unconscious as an affective core**: Though lacking cognition, the unconscious is powerful and deterministic. People can learn to modulate intense affective experiences of the unconscious through the experience of care, leading to an integration of affect and cognition.
- **Appreciate the affective system and its triggers:** The affective core can be triggered, and in the absence of adequate cognitive modulation, this can lead to experiences that feel both alien and powerful. Caregivers can help modulate these experiences, leading to genuine integration of affect and cognition.
- **Recognize the modulating role of the therapist:** Therapists can help patients recognize and digest overwhelming affective experiences. This process can be challenging and requires the therapist to maintain their capacity to think in order to guide both through the experience. This demands a curiosity and willingness to recognize the powerful, uncontrollable parts of oneself. In doing so we facilitate the integration of affect and cognition.

## Data Availability

Data sharing not applicable to this article as no datasets were generated or analysed during the current study.
